# The effect of patient participation on trust in primary health care physicians among patients with chronic diseases: the mediating role of perceived value

**DOI:** 10.3389/fpubh.2025.1586123

**Published:** 2025-05-14

**Authors:** Li Zhang, Baokai Wang, Chang Fu

**Affiliations:** ^1^School of Health Management, Binzhou Medical University, Yantai, Shandong, China; ^2^Yantai Yuhuangding Hospital, Yantai, Shandong, China

**Keywords:** patient participation, perceived value, patient trust, chronic disease, primary health care

## Abstract

**Background:**

Low levels of trust in primary health care physicians (PHCPs) significantly decrease primary health care utilization among patients with chronic diseases. In the context of the Chinese government having made considerable efforts to strengthen the development of chronic disease management within Chinese primary health care institutions, it is of great theoretical and practical significance to investigate ways to improve the trust in PHCPs among patients with chronic diseases. This study aimed to explore the impact of patient participation on patient trust in PHCPs, and to examine the mediating role of patient perceived value on this relationship.

**Methods:**

This study included 884 patients with chronic diseases from a cross-sectional survey conducted in Shandong Province, China, from July to August 2023, using a multistage stratified sampling method. Structural equation modeling was conducted to investigate the relationships between patient participation, patient perceived value, and patient trust.

**Results:**

Patient participation had a direct effect on patient trust (Bias-corrected 95%CI = 0.10–0.20; Percentile 95%CI = 0.10–0.20). Furthermore, patient perceived value mediated the relationship between patient participation and patient trust (Bias-corrected 95%CI = 0.15–0.32; Percentile 95%CI = 0.15–0.32).

**Conclusion:**

Patient participation cannot only directly and positively predict patient trust in PHCPs, but also indirectly affect patient trust through patient perceived value. These findings highlight the importance of patient participation and perceived value to improve the trust in PHCPs among patients with chronic diseases. Therefore, health managers and policymakers should facilitate patient participation and recognize patient perceived value during the service delivery process.

## 1 Introduction

Chronic diseases pose a considerable threat to human wellbeing and have emerged as a prominent public health issue worldwide, owing to their high prevalence and related mortality (1). With the persistent advancement of industrialization, urbanization, and population aging in China, the prevalence of chronic diseases has increased significantly and has trended upward annually (2, 3). This phenomenon has generated a growing burden on individuals, families, society, and the healthcare system (4, 5). In this context, effective management of chronic diseases has become a primary focus because of its connection to the accessibility of convenient, affordable, and appropriate health services. International experience has consistently demonstrated that primary health care facilities serve as the optimal foundation for implementing comprehensive chronic disease management (6–8). Since 2009, China’s health system reform has strategically integrated chronic disease management into its essential public health services, which are widely accessible to residents, and mainly delivered by primary health care institutions (9).

Primary health care is acknowledged as the foundation and ultimate solution for chronic disease prevention and control in China (10). However, China lacks rigorous enforcement of a referral system, which allows individuals to directly access higher-tier hospitals without prior primary care consultation (11). Furthermore, over the past decades, there have been persistent disparities in the allocation of medical resources including health care facilities, workforce, and technology in China. These disparities have severely constrained the capacity of primary health care institutions to provide comprehensive clinical and preventive care (12). As a result, this has created distrust in primary health care physicians (PHCPs) among patients with chronic diseases (13). Therefore, in China, many patients with chronic diseases prefer to seek basic medical services at secondary or tertiary hospitals, rather than opting for primary health care institutions, which overcrowding high-level hospitals while weakening primary health care institutions (14, 15). Additionally, this trend exacerbates the economic burden on patients with chronic diseases (16), wastes limited medical resources (17), and ultimately threatens the efficiency of China’s healthcare system (18).

In response, China has substantially increased financial investment and implemented beneficial programs and policies aimed at reinforcing its primary health care system, which bears the primary responsibility for comprehensive chronic disease prevention and management (19). One previous empirical study has revealed that chronic disease management within Chinese primary health care institutions can leverage current resources to enhance primary health service utilization, contain health expenditure growth, and minimize unnecessary hospital admissions (20). Furthermore, such management models do not adversely affect and potentially improve patients’ intermediate- and long-term health outcomes (20). However, lower levels of trust in PHCPs significantly decrease primary health service utilization among patients with chronic diseases (21). The Chinese government is currently making considerable efforts to strengthen the development of chronic disease management within Chinese primary health care institutions. In this context, investigating ways to improve trust in PHCPs among patients with chronic diseases is of great theoretical and practical significance for the development of chronic disease management and primary health care.

Trust, a major driving force in human relationships, is increasingly recognized as the keystone determinant of the doctor–patient relationship and the effectiveness of health service provision (22–24). Trust in the primary health care field has garnered increasing attention in China because patient trust in PHCPs has been reported to have consistently declined over the past few decades (25). Patient trust represents the patient’s belief that the physician prioritizes his needs, interests, and wellbeing, and will provide them with proper medical and health services (26). It symbolizes the patient’s faith in the received service, where the patient believes that physicians are competent and reliable in delivering the requisite standard of care (27). Considerable evidence has verified that patient trust is a critical facilitator in fostering patient’s adherence to treatment (28, 29), improving self-care (30), better control of chronic conditions, health outcomes, and wellbeing (22, 23, 31, 32), promoting better utilization of health services (33), enhancing patient satisfaction (34), and increasing patient revisit intention (35). Given the crucial significance of patient trust, it is imperative to thoroughly explore the antecedents and mechanisms of trust in PHCPs among patients with chronic diseases.

Patient trust is influenced by multiple factors, encompassing social background, medical circumstances, and personal attributes of both doctors and patients (36). Given the diverse, complex, and persistent needs of patients with chronic diseases, fostering patient involvement in health care and enabling health care providers to comprehend individual patient requirements, and devise appropriate solutions to meet these needs are especially significant aspects (4). Moreover, within the framework of the patient-centered service model, the dynamic between patients and doctors has transformed into a collaborative partnership that places significant emphasis on self-care and mutual participation (37). This approach upholds the principle of respecting patient autonomy, fostering an environment where medical information and care-related decisions are arrived at through shared decision between health care providers and patients (38). Notably, with the growing prevalence of chronic diseases, patient participation in health care encounter has emerged as a pivotal factor in realizing the goals of patient-centered care and enhancing chronic disease management (39–41). Consequently, a general consensus currently exists that patients with chronic diseases ought to actively participate in treatment, and the participation among patients suffering from chronic diseases is now garnering more attention than ever before (38).

Patient participation is characterized in a broader sense, including sharing information, expressing treatment preferences, opinions, and experiences, participating in decision-making processes and self-management, and providing suggestions (42, 43). When patients and doctors engage in thorough discussions about treatment choices and possible risks, patients are more likely to clearly understand their care plans, develop realistic expectations about treatment outcomes, effectively handle uncertainties, and, as a result, build greater trust in their doctors (44). In addition, the improved health literacy and self-care competencies of patients through their engagement in medical visits allow them to feel more active and confident about their health conditions, which serves as critical mediators for establishing and maintaining trust in physicians (45). Previous study showed that effective communication about treatment between PHCPs and patients can increase patient trust in PHCPs by improving the continuity and interactivity of health service delivery (27). Moreover, patients who actively participate in their own health care decision-making tend to exhibit a higher level of trust in their PHCPs (46). Therefore, we propose Hypothesis 1 (H1): patient participation may positively influence patient trust in PHCPs.

While H1 establishes the direct relationship between patient participation and patient trust, the mechanisms underlying this association remain underexplored. To address this gap, we draw on the co-creation framework of health service delivery, which posits that health care is a unique profession in which doctors and patients are major participants who co-create the process of service delivery (38). Patient participation commonly occurs during encounters with health care providers, in which patients participate in diverse activities (4). Patient involvement in co-creation of health services can create value related to experience, learning, relationships, and psychological benefits, and reduce risk perception, which may lead patients to weigh the benefits over the costs (47, 48). The Theory of Perceived Value indicates that positive disconfirmation arises, leading to perceived value, when the perception of service benefits surpasses the associated costs (49). Research based on the Theory of Reasoned Action indicates that evaluation of behavioral outcomes can significantly shape customer attitudes (50). When patients perceive the value of the services they receive, their uncertainty during health care visits is reduced and their likelihood of relying on and trusting doctors increases (51, 52). One previous Chinese study has shown that patient perceived value has a beneficial effect on patient trust in PHCPs (53). Drawing upon the aforementioned literature review, we therefore propose Hypothesis 2 (H2): patient perceived value may mediate the relationship between patient participation and patient trust in PHCPs.

Given the pivotal role of patient trust in managing chronic diseases within primary health care institutions, exploring pathways to enhance trust in PHCPs is imperative. In addition, despite the widespread recognition of patient-centered services and patient participation as crucial to current views of the optimal physician-patient relationship, the influence of patient participation on patient trust in PHCPs remains underexplored. Furthermore, the mediating effect of patient perceived value on the relationship between patient participation and patient trust in PHCPs has seldom been investigated. The primary objectives of our study were to examine the relationship between patient participation and patient trust in PHCPs, and to explore the mediating role of patient perceived value in shaping this relationship. This would aid in understanding the significance of patient participation in fostering patient trust in PHCPs. Furthermore, our findings would provide implications for health managers to promote patient participation, and recognize patients’ values based on their perceptions and experiences throughout the health care visit process.

## 2 Materials and methods

### 2.1 Study design and participants

This study was conducted as part of a project supported by the Natural Science Foundation of Shandong Province (No. ZR2022QG090), which investigates the effects of patient participation and patient experiences (e.g., perceived value and patient trust) on patient loyalty across Shandong Province. The current analysis focuses specifically on the patients with chronic diseases. This cross-sectional survey of the project was conducted from July to August 2023 in Shandong Province, which has 16 prefectures, and is located in eastern China, representing a characteristic Chinese area with advanced economic development and high population density. A multi-stage stratified sampling method was adopted to select participants. First, two counties or districts were randomly selected from each prefecture. Second, within each county or district, we implemented a stratified sampling approach by classifying areas into urban and rural categories, and subsequently randomly selecting one urban subdistrict and one rural township from each county or district. Third, we randomly selected one township hospital and one community health service center in each rural township or urban subdistrict. Ultimately, our study included 64 primary health care institutions as research sites. We used a convenience sampling method for patient recruitment to collect data. All surveys were carried out through in-person interviews conducted by postgraduate and undergraduate students who received training from researchers at the School of Health Management, Binzhou Medical University.

Patients were eligible to participate if they were 18 years or older, had no cognitive disability, and had utilized health or medical services at the study sites before the survey. Following a comprehensive explanation of the research purpose, these patients were recruited for the survey and assured of receiving a small gift of appreciation after successfully completing the questionnaire. The following exclusion criteria were applied: (1) patients suffering from communication or cognitive disabilities, and (2) inability to comprehend the questionnaire content. Individuals willing to participate in the survey provided written informed consent, and were notified that they had the right to withdraw from their participation at any stage. All collected study data were anonymized and remain confidential. In this study, 16 variables including 8 socio-demographic characteristics and 8 latent variables were assessed. Benter and Chou indicated that the sample size should be 10 times the number of variables in the analysis, yielding an initial estimate of 160 participants. Considering an expected 80% effective response rate, the adjusted minimum sample size was set at 200 (54). The sample size of this study was 884, which met the recommended requirement. Hence, our study employed the sample size large enough to ensure robust statistical power. The Ethics Committee of Binzhou Medical University approved this study (No. 2021-337).

### 2.2 Measures

#### 2.2.1 Patient participation in health service visits

Patient participation in health service visits was evaluated utilizing the Perceived Involvement in Care Scale (PICS) (55). The PICS uses 13 items to evaluate patients’ perspectives on their engagement in accessing symptom related details, communicating their concerns to PHCPs, and involving them in treatment decision-making during medical consultations. Previous research has demonstrated that the PICS has good reliability and validity, and is appropriate for use among Chinese people (4, 38). The response options are scored dichotomously, with “agree” being awarded 1 point and “disagree” being awarded 0 points. The potential scores on the scale vary from 0 to 13, with higher scores reflecting a higher degree of patient participation during health care visits. In this study, the Cronbach’s *α* for this scale was 0.82.

#### 2.2.2 Patient perceived value

The adapted Chinese version of the Patient Perceived Value Scale was adopted to evaluate patients’ perceptions of primary health service value (56). This scale has 10 items and comprises of three dimensions, which are functional value, emotional value, and social value. Items are rated on a five-point Likert scale, ranging from 1 (“extremely disagree”) to 5 (“extremely agree”). The mean value of these 10 items was used for data analysis, with higher scores indicating greater levels of patient perceived value. The Cronbach’s *α* for this scale was 0.95.

#### 2.2.3 Patient trust

The Chinese version of the Wake Forest Physician Trust Scale (WFPTS) was used to assess patients’ trust in PHCPs (57). The Chinese WFPTS contains 10 items and consists of two constructs: technical competence and benevolence. Responses are reported on a five-point Likert scale, ranging from one (“strongly disagree”) to five (“strongly agree”). The 10 items are averaged to create a single scale, with higher scores signifying a greater level of patient trust in PHCPs. In the present study, the Cronbach’s *α* for this scale was 0.88.

### 2.3 Statistical analysis

Statistical analyses were performed using the IBM SPSS 24.0 version program and Amos 21.0 software. Descriptive statistics were employed to generate a comprehensive overview of participants’ characteristics and the study variables. Common method bias that may exist in self-reported data was assessed through Harman’s single factor test. The presence of significant multi-collinearity was tested using a variance inflation factor (VIF) threshold of 5 and a tolerance value of 0.1 as diagnostic criteria (58). Path analysis via structural equation modeling (SEM) was conducted to investigate the hypothesized relationships among patient participation, patient perceived value, and patient trust. Acceptable goodness of fit verified when *χ*^2^/df was less than 5, Comparative Fit Index (CFI), Normed Fit Index (NFI) and Tucker Lewis Index (TLI) were above 0.9, and the Root Mean Square Error of Approximation (RMSEA) was less than 0.08 (38). The mediation effect was verified using bootstrapping method with 5,000 random samples drawn from the dataset. Statistical significance was determined using Percentile 95% confidence intervals (CI) and Bias-corrected 95%CI calculations. Statistical significance was established if the CI did not include the value of zero.

## 3 Results

### 3.1 Participants’ sociodemographic characteristics

**Figure 1:**
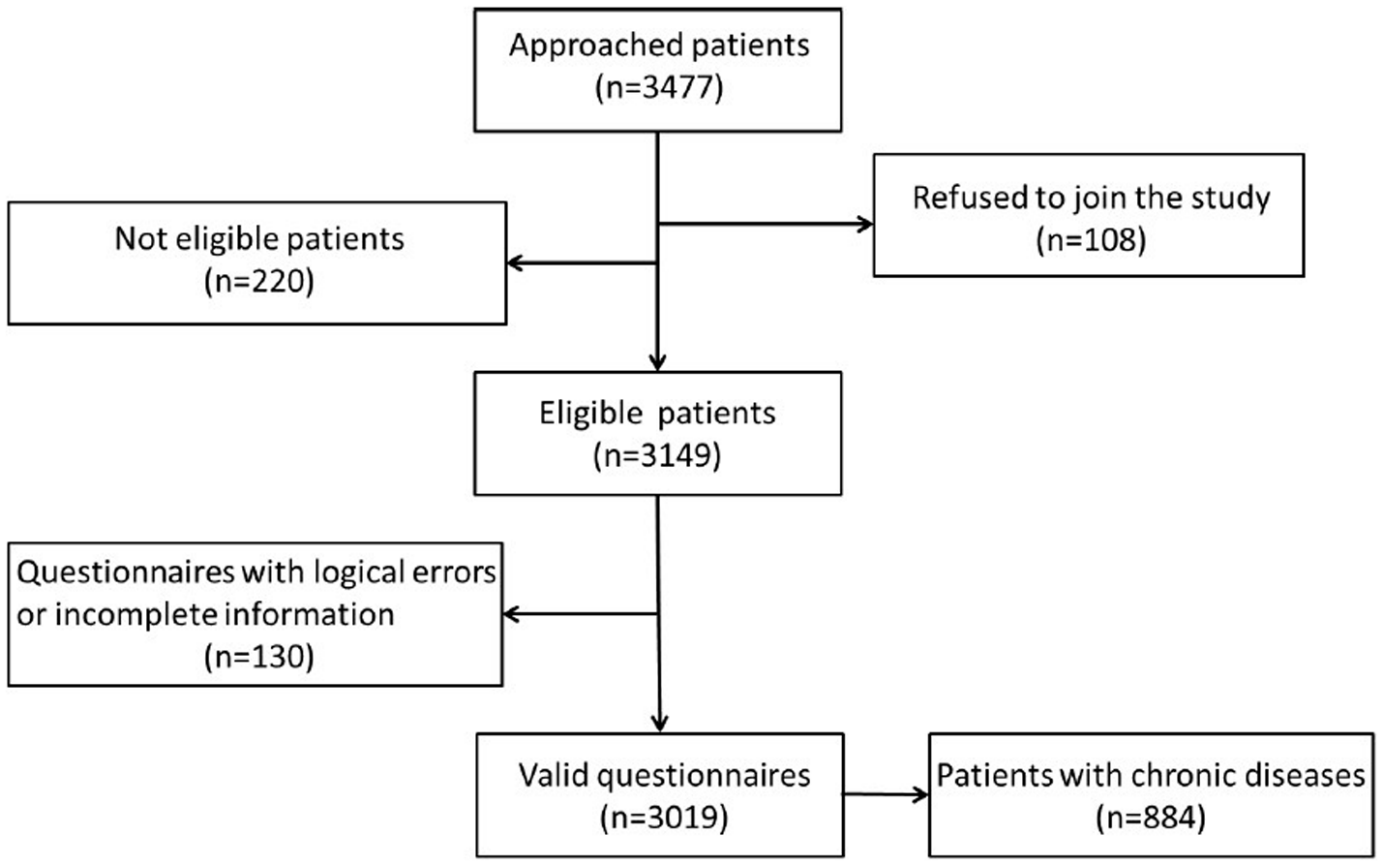
Patient flow diagram showing the study participation selection.

We invited a total of 3,477 patients to participate in the survey, 108 patients were rejected, and 220 patients were ineligible because they had never before visited the study institutions. This resulted in a sample of 3,149 respondents (90.57%) providing information. We excluded 130 questionnaires owing to logical errors or incomplete information. Thus, we finally collected 3,019 responses, with a valid questionnaire response rate of 95.87%. We filtered 884 valid questionnaires in which patients had a chronic disease based on participants’ responses to the question about whether they suffered from a chronic disease, comprising 29.28% of the overall sample. See Figure 1 for details.

Table 1 reports the sociodemographic characteristics of the 884 participants in our study, which comprised 487 females and 397 males, with an average age of 59.47 ± 14.24 years. Among them, more than two thirds (77.70%) were rural, and nearly half were single, divorced, or widowed (49.21%). In terms of occupation status, 362 participants were employed. Regarding educational level, 87.22% of participants had attained senior high school or below education at survey. Patients with a per capita monthly income of 3,000 RMB or less occupied 73.08% (*n* = 646). Of these, only 6 patients reported having no medical insurance.

**Table 1:** Socio-demographic characteristics of the participants.

Characteristic		*n*	%
Gender	Male	397	44.91
	Female	487	55.09
Age	(Mean ± SD)	59.47 ± 14.24	
	<45 years	113	12.78
	45–59 years	322	36.43
	≥60 years	449	50.79
Residence	Rural	678	77.70
	Urban	206	23.30
Marital status	Married	449	50.79
	Single/divorced/widowed	435	49.21
Occupation status	Employed	362	40.95
	Retired/unemployed	522	59.05
Educational level	Senior high school or below	771	87.22
	College or above	113	12.78
Personal monthly income	≤3,000 RMB	646	73.08
	>3,000 RMB	238	26.92
Medical insurance	No	6	0.68
	Urban Employee Basic Medical Insurance	146	16.52
	Urban and Rural Residents Basic Medical Insurance	690	78.05
	Other medical insurance	42	4.75

SD, Standard deviation; 1 RMB = 0.14 US dollars (the date of conversion: April 30, 2025).

### 3.2 Testing for common method bias and multi-collinearity

Harman’s single factor test identified six common factors with eigenvalues exceeding 1. The percentage of variance attributed to the first common factor was 35.09%, falling below the suggested cutoff of 50% (58). Therefore, the issue of common method bias was not significant in this research.

**Figure 2:**
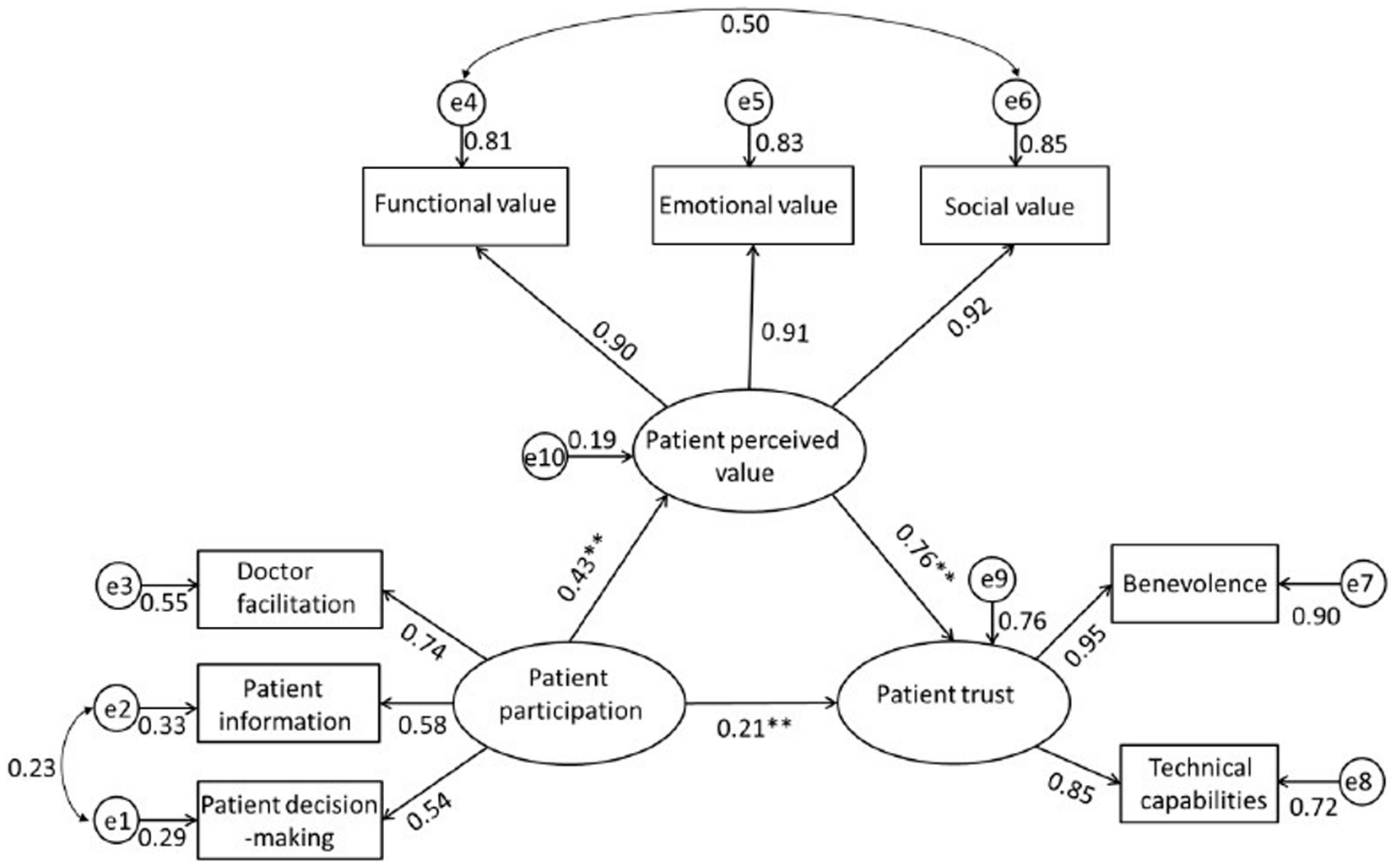
Path diagram derived from structural equation modeling illustrating the hypothesized mediating effect of patient perceived value on the relationship between patient participation and patient trust. The arrows represent significant relationships between two variables. The value next to the arrow represents the direct effect *β* standardized path coefficient (for indirect effect value see Table 3). *e* represents the error and the number next to it is the standardize relationship. **p < 0.01.

Multi-collinearity diagnostics presented the ranges of the tolerance and VIF were 0.20–0.71 and 1.41–4.98, respectively, both falling within acceptable limits. We, therefore, concluded that there was not present multi-collinearity problem in this analysis (Figure 2).

### 3.3 Descriptive analysis and correlations of the main study variables

Table 2 lists all mean scores, standard deviations, and the degree of correlation of the study constructs. All study variables were significantly and positively correlated with each other at the significance level of 0.001.

**Table 2:** Correlation between latent variables.

Variables	1	2	3	4	5	6	7	8
1. Emotional value	1.00							
2. Social value	0.84**	1.00						
3. Functional value	0.82**	0.75**	1.00					
4. Technical capabilities	0.66**	0.67**	0.65**	1.00				
5. Benevolence	0.73**	0.74**	0.73**	0.80**	1.00			
6. Doctor facilitation	0.29**	0.30**	0.29**	0.34**	0.38**	1.00		
7. Patient information	0.23**	0.23**	0.23**	0.26**	0.29**	0.43**	1.00	
8. Patient decision-making	0.21**	0.22**	0.21**	0.25**	0.28**	0.40**	0.47**	1.00
Mean	3.91	3.93	3.81	3.69	3.72	4.40	3.48	2.77
SD	0.54	0.57	0.60	0.54	0.55	1.09	0.99	1.39

SD, Standard deviation. **p < 0.01.

### 3.4 Mediating effect of patient perceived value on the relationship between patient participation and patient trust in PHCPs

Consistent with the hypothetical theory framework, we performed a path analysis via SEM to verify the relationship between patient participation and patient trust in PHCPs, and the mediating effect of patient perceived value on the relationship between them. As shown in Figure 1, the hypothesized model fits the data reasonably well: *χ*^2^/df = 4.969, NFI = 0.984, CFI = 0.987, TLI = 0.976, RMSEA = 0.067. The findings presented in Table 3 suggest that patient participation has a direct and positive correlation to patient trust in PHCPs (*β* = 0.21) and patient perceived value (*β* = 0.43). Patient perceived value was significantly associated with patient trust in PHCPs (*β* = 0.76).

**Table 3:** Path coefficient results in structural equation model.

Path				Standard coefficient	Unstandard coefficient	S.E.	*t* value
a	Patient participation	→	Patient perceived value	0.43	0.31	0.04	7.99**
b	Patient perceived value	→	Patient trust	0.76	0.73	0.03	25.92**
c	Patient participation	→	Patient trust	0.21	0.15	0.02	6.51**

SE, Standard error. **p < 0.01.

Table 4 displays that patient participation directly influences patient trust in PHCPs (Bias-corrected 95%CI = 0.10–0.20; Percentile 95%CI = 0.10–0.20). It also reveals a notable indirect impact of patient perceived value on the relationship between patient participation and patient trust in PHCPs (Bias-corrected 95%CI = 0.15–0.32; Percentile 95%CI = 0.15–0.32), the medicating effect was 0.23, which supported Hypotheses 2. The aggregate impact of patient participation on patient trust in PHCPs comprised both the indirect and direct effects, which was 0.38. The direct effect accounted for 39.47%; the mediation effect of patient perceived value occupied 60.53%.

**Table 4:** Total, direct, and indirect effects of patient participation on patient trust.

Route	Point estimate	Product of coefficients		Bootstrapping			
				Bias-corrected 95%CI		Percentile 95%CI	
		SE	*Z*	Lower	Upper	Lower	Upper
Direct effect							
H1: Patient participation → patient trust	0.15	0.03	5.00	0.10	0.20	0.10	0.20
Indirect effect							
H2: Patient participation → patient perceived value → patient trust	0.23	0.04	5.75	0.15	0.32	0.15	0.32
Total effect							
Patient participation → patient trust	0.38	0.05	7.60	0.28	0.48	0.28	0.48

SE, Standard error.

## 4 Discussion

This study implemented a pathway mediation model linking patient participation to patient trust in PHCPs through patient perceived value by adopting a cross-sectional survey of 884 patients with chronic diseases from 64 primary health care institutions in eastern China. The results showed that patient participation cannot only directly and positively predict patient trust in PHCPs, but also indirectly affect patient trust in PHCPs through patient perceived value. Our findings reveal that patient participation exerts a favorable influence on patient trust in PHCPs, and highlight the critical role of patient perceived value in this relationship. To our knowledge, this study represents one of the first empirical investigations to explore the mediating role of patient perceived value in the relationship between patient participation and patient trust in PHCPs. Our research results are anticipated to offer insights for health managers and policy makers to attach great importance to patient participation and perceived value during health care visits to improve patient trust in PHCPs.

For the two dimensions of patient trust in PHCPs, the score for “benevolence” (3.72 ± 0.55) was higher than for “technical capabilities” (3.69 ± 0.54), which is consistent with other research showing that patients have greater trust in PHCPs’ benevolence than in their technical capabilities (27). Our study results indicated that the average score of patient trust in PHCPs was 3.71 ± 0.52, which is similar to the finding of a previous study (3.70) conducted in primary health care institutions across eastern, central, and western China, but higher than the findings from China’s northeastern province (3.39) (4). This discrepancy may be related to geographic and economic factors that influence the service provision capacity of primary health care facilities. In addition, it found that trust in PHCPs among Chinese patients remained relatively low compared to that in large hospitals (36, 45). Compared to large hospitals, primary health care institutions have long had a serious shortage of resources, workforce, and technology, which has resulted patients lower trust in PHCPs. The global health paradigm increasingly positions patient trust as a key requirement for the achievement of health initiatives (59), however, low levels of patient trust in PHCPs remains a prominent issue in the Chinese healthcare system (31, 60). To resolve this problem, primary health care managers should prioritize strategies aimed at enhancing patient trust.

The analysis results confirmed Hypothesis 1 (H1), namely, patient participation was positively correlated with patient trust in PHCPs, meaning that the higher the level of participation by patients with chronic diseases in health care visits, the more likely they are to trust PHCPs. To date, few studies have explored the factors of patient trust in PHCPs from the perspective of patient participation, despite the widespread acknowledgment of patient involvement as a cornerstone of patient-centered care for individuals with chronic diseases (61). Our results add empirical evidence to the literature on the importance of participation by patients with chronic diseases in enhancing their trust in PHCPs. When patients with chronic diseases actively engage during the process of health care delivery, their health requirements, personal preferences, and recommendations will probably be integrated into health service designs following comprehensive discussions with their PHCPs (38). This participatory approach significantly increases the likelihood that the service will meet their expectations (27). Furthermore, patients and doctors shared that active and mutual participation in the service process can eliminate patients’ misunderstandings, improve their recognition and cooperation in decision-making, and decrease their perceptions of complexity and uncertainty regarding treatment choices and possible risks (45, 46). This tendency ultimately fosters a trusting and respectful patient-physician relationship (36). Our findings demonstrate that active participation by patients with chronic diseases significantly enhances their trust in PHCPs. Therefore, health managers should promote and popularize the shared decision-making, and train PHCPs to develop skills that actively encourage patients with chronic diseases to ask questions and voice their concerns, thereby fostering greater patient involvement in their own care processes.

The results of the study confirmed that patient perceived value served as a significant mediator in the pathway linking patient participation and patient trust, thereby confirming Hypothesis 2 (H2). Although a previous study extensively examined customer involvement as a driver of customer value within the marketing environment (48), the relationship between patient participation and patient perceived value remains underexplored in health services. Our study provides a groundbreaking empirical insight that active participation in health service visits among patients with chronic diseases could significantly increase their perceived value in the primary health care sector. Health care providers should create an atmosphere in which patients with chronic diseases participate actively in their service delivery. Additionally, our findings align with prior empirical research, which has consistently demonstrated that patient perceived value positively influences patient trust in health care providers (52, 53), which implies a crucial role of perceived value in enhancing patient trust. Therefore, primary health care administrators ought to prioritize the core values that patients care about, and strive to provide health services that are beneficial and tailored to patients’ requirements. For example, they can holistically optimize the service process and continuously improve the service quality, thereby improving patient perceived value.

Although our study offers both theoretical insights and practical applications, it is not without its limitations. Firstly, similar to other studies using a cross-sectional design, the present study has limitations in inferring any causal relationships between the investigated variables. Future research could adopt longitudinal designs or experimental methods to explore causality. Secondly, our study was conducted only in Shandong province of China owing to time, funding, and human resource constraints, which might restrict the generalizability of our findings. Future studies could broaden the sample to diverse regions or even different nations to enhance the universality of the research conclusions. Thirdly, as the survey data were self-perceived or self-reported, there was potential information or recall bias, although the interviewers were trained in methods to aid patients in providing precise answers. Fourthly, our study only sheds light on the mediating role of patient perceived value in investigating the relationship between patient participation and patient trust, future research is needed to focus on the moderating or mediating effects of other situational factors to better understand the mechanism. Finally, patient trust in PHCPs is complicated, so it is relatively simple to build the model only from the perspective of patient participation in this paper, without considering other influencing factors. Other determinants should be explored in the future research to provide more robust results to build the model.

## 5 Conclusion

This study explored the pathways linking patient participation and trust in PHCPs among patients with chronic diseases. These findings imply that patient participation is directly associated with patient trust, and indirectly associated through patient perceived value. These findings contribute to the few literatures exploring the effect of patient participation on patient trust, and highlight the importance of patient perceived value in primary health care. The results also offer valuable implications for health managers and policymakers facilitating patient participation and recognizing patient perceived value for improving the trust in PHCPs among patients with chronic diseases. Therefore, to foster greater patient trust in PHCPs, health managers should promote and popularize the shared decision-making, and train PHCPs to develop skills to facilitate patient participation. Additionally, primary health care administrators should prioritize patient value and strive to provide tailored and beneficial health services by optimizing processes and improving service quality.

## Data Availability

The raw data supporting the conclusions of this article will be made available by the authors without undue reservation.
